# CDKL5 localizes at the centrosome and midbody and is required for faithful cell division

**DOI:** 10.1038/s41598-017-05875-z

**Published:** 2017-07-24

**Authors:** Isabella Barbiero, Davide Valente, Chetan Chandola, Fiorenza Magi, Anna Bergo, Laura Monteonofrio, Marco Tramarin, Maria Fazzari, Silvia Soddu, Nicoletta Landsberger, Cinzia Rinaldo, Charlotte Kilstrup-Nielsen

**Affiliations:** 1 Department of Biotechnology and Life Sciences, University of Insubria, 21052 Busto Arsizio, Italy; 2 Unit of Cellular Networks and Molecular Therapeutic Targets, Department of Research, Advanced Diagnostic, and Technological Innovation, Regina Elena National Cancer Institute – IRCCS, 00144 Rome, Italy; 3 Institute of Molecular Biology and Pathology (IBPM), National Research Council (CNR), c/o Sapienza University, 00185 Rome, Italy; 4 Department of Medical Biotechnology and Translational Medicine, University of Milan, 20090 Segrate, Italy; 5 Centre for Drug Research, Division of Pharmaceutical Biosciences, University of Helsinki, Helsinki, Finland; 6 Laboratory of Biomedical Research “Fondazione Niccolò Cusano per la Ricerca Medico-Scientifica”, Niccolò Cusano University, Rome, Italy

## Abstract

The cyclin-dependent kinase-like 5 (*CDKL5*) gene has been associated with rare neurodevelopmental disorders characterized by the early onset of seizures and intellectual disability. The CDKL5 protein is widely expressed in most tissues and cells with both nuclear and cytoplasmic localization. In post-mitotic neurons CDKL5 is mainly involved in dendritic arborization, axon outgrowth, and spine formation while in proliferating cells its function is still largely unknown. Here, we report that CDKL5 localizes at the centrosome and at the midbody in proliferating cells. Acute inactivation of CDKL5 by RNA interference (RNAi) leads to multipolar spindle formation, cytokinesis failure and centrosome accumulation. At the molecular level, we observed that, among the several midbody components we analyzed, midbodies of CDKL5-depleted cells were devoid of HIPK2 and its cytokinesis target, the extrachromosomal histone H2B phosphorylated at S14. Of relevance, expression of the phosphomimetic mutant H2B-S14D, which is capable of overcoming cytokinesis failure in HIPK2-defective cells, was sufficient to rescue spindle multipolarity in CDKL5-depleted cells. Taken together, these results highlight a hitherto unknown role of CDKL5 in regulating faithful cell division by guaranteeing proper HIPK2/H2B functions at the midbody.

## Introduction

CDKL5 is a serine-threonine kinase that was identified through a transcriptional mapping study aimed at identifying disease causing genes in the Xp22 region^1^. The subsequent identification of mutations in the *CDKL5* gene in patients with the Hanefeld variant of Rett syndrome or early infantile epileptic encephalopathy (OMIM #300672) suggested the involvement of this gene in human brain functions^2^. In agreement, the two existing *Cdkl5* knock-out mouse models are characterized by impaired learning and memory, autistic-like features, and motor deficits reconciling several aspects of the clinical spectrum present in patients with mutations in *CDKL5* ^3, 4^.

The *CDKL5/Cdkl5* gene is widely transcribed and the protein can be detected in most tissues and cells, both in the nucleus and in the cytoplasm^5, 6^. However, CDKL5 expression reaches highest levels in the brain^6^ and because of the evident brain-associated functions, most studies have focused on the neuronal roles of CDKL5. In the brain CDKL5 expression is low at embryonic stages but a significant induction can be observed in the neuronal compartment in the first post-natal days supporting a role during neuronal maturation^5^. In post-mitotic neurons the levels and distribution of CDKL5 are regulated by neuronal activity indicating that the protein responds promptly to external stimuli^7, 8^. Of relevance, neurons devoid of the kinase are characterized by defects in axon formation and outgrowth, dendritic arborization, spine morphology, and synaptic transmission, underscoring the importance of CDKL5 for brain development and functioning^4, 6, 9, 10^.

While the functions of CDKL5 in post-mitotic neurons are under continuous investigation, its role in proliferating cells is still largely unknown. CDKL5 overexpression induces cell cycle arrest in neuroblastoma cells^11^ whereas CDKL5 inhibition, by RNAi or targeted gene disruption, was shown to increase bromodexoyuridine incorporation^11, 12^. Although these data suggest the involvement of CDKL5 in cell proliferation, no information is available regarding the functions and the subcellular localization of the kinase during the cell cycle. In the current study we examined the localization of CDKL5 in interphase, mitosis, and cytokinesis of proliferating cells. Besides the typical nuclear punctuate staining of CDKL5 in interphase cells^13^, we also found CDKL5 to be localized at the centrosomes and at the midbody.

In animal cells, centrosomes form when a pair of orthogonally positioned centrioles assemble and organize a matrix of proteinaceous pericentriolar material around themselves. Centrioles act as the centrosome organizer and their duplication controls centrosome number. Like DNA, centrioles duplicate semi-conservatively exactly once per cell cycle^14^. The centrosome serves as the main microtubule-organizing center that contributes to cell adhesion, motility, and polarity in interphase and to bipolar spindle formation and timely mitotic progression in mitosis^15, 16^. During mitosis, the presence of two centrosomes per cell ensures the bipolar nature of the spindle and the equal segregation of chromosomes to two daughter cells. Quantitative or qualitative centrosome defects may lead to multipolar spindle formation and, eventually, loss of mitotic fidelity and acquisition of chromosome instability^17, 18^.

The midbody is the narrow intercellular bridge containing bundles of microtubules derived from the mitotic spindle that connects the two daughter cells in cytokinesis. A complex network of components impacting on vesicle and membrane trafficking, cytoskeleton, chromosomes, cell cycle and lipid rafts affects midbody formation and cleavage^19^. Among the numerous midbody components, we have shown that HIPK2, an evolutionary conserved kinase whose large number of substrates includes the Rett syndrome associated factor MeCP2^20^, localizes at the midbody and is required for faithful cytokinesis^21^. HIPK2 contributes to abscission, the last step of cell division, by phosphorylating extrachromosomal histone H2B at serine 14 (S14) at the midbody. In HIPK2-defective cells, expression of a phosphomimetic H2B-S14D mutant overcomes the cytokinesis failure^21^.

By biochemical and functional assays, we confirmed the presence of CDKL5 both at centrosomes and at the midbody and highlighted the involvement of CDKL5 in cell division through the regulation of HIPK2/H2B functions.

## Results

### CDKL5 localizes at the centrosome and midbody

**Figure 1:**
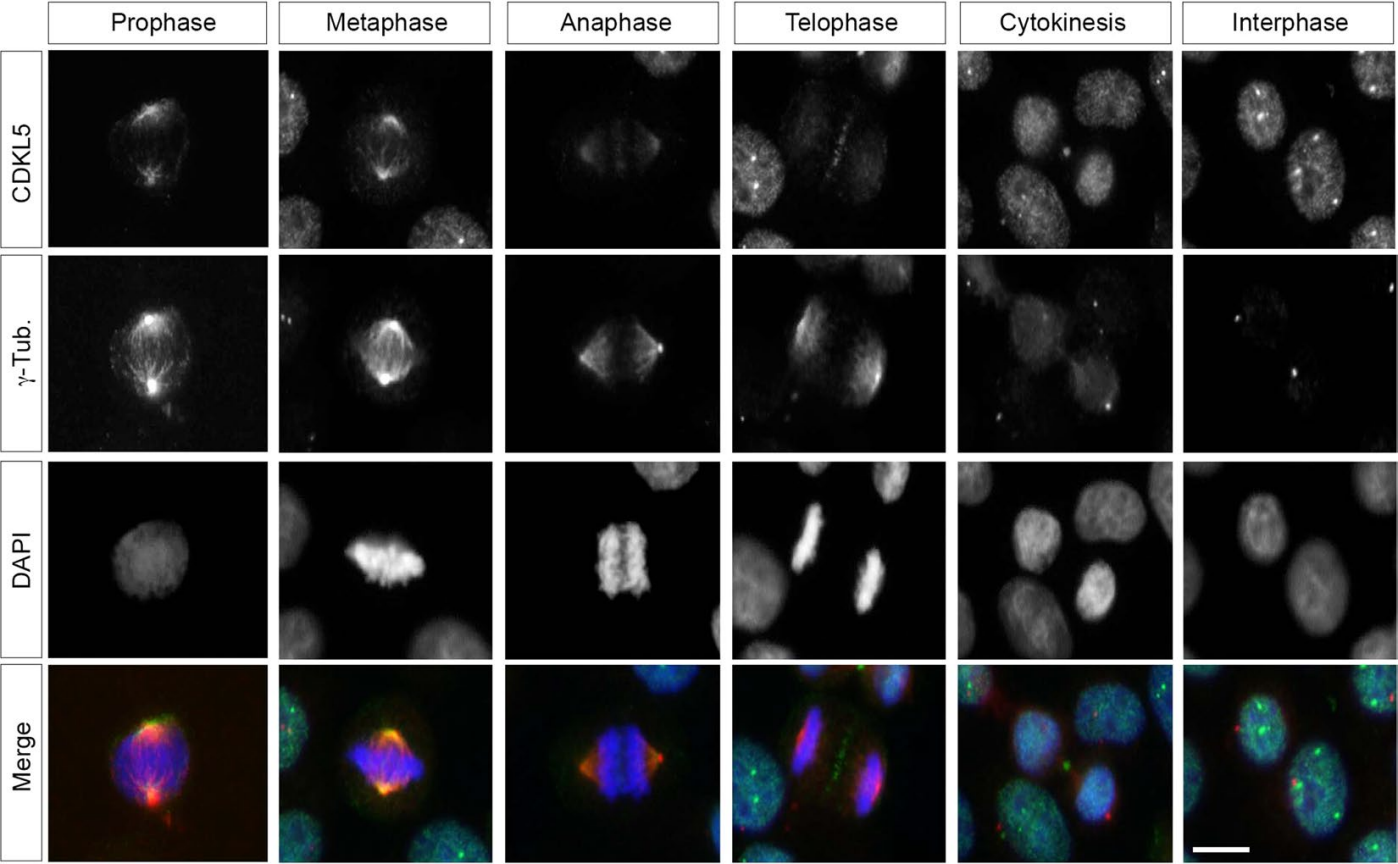
CDKL5 localizes at the centrosome and at the midbody in proliferating cells. HeLa cells were stained with Abs against CDKL5 (polyclonal, green) and γ-tubulin (red) and, to visualize DNA, with DAPI (blue). Scale bar, 10 µm.

To investigate the function(s) of the ubiquitously expressed CDKL5 in proliferating cells we started evaluating the subcellular localization of the kinase during the cell cycle. The distribution of endogenous CDKL5 was analyzed in HeLa cells by immunofluorescence (IF) during interphase, mitosis, and cytokinesis (Fig. 1). We observed a quite dynamic localization of CDKL5 at different mitotic and cytokinetic subcompartments. In prophase and metaphase, CDKL5 is detectable at the mitotic spindle poles where it colocalizes with the centrosomal marker γ-tubulin. As cells progress in telophase, CDKL5 is no longer detectable at the centrosome but localizes at the midzone. In the subsequent steps of cytokinesis CDKL5 is clearly detectable at the midbody, where it remains during abscission. As expected, in interphase we observed the typical punctuate nuclear staining of CDKL5, which corresponds to nuclear speckles enriched in mRNA splicing factors^13^.

Next, through additional studies we validated the centrosomal and midbody localization of CDKL5 suggested by the above IF results.

**Figure 2:**
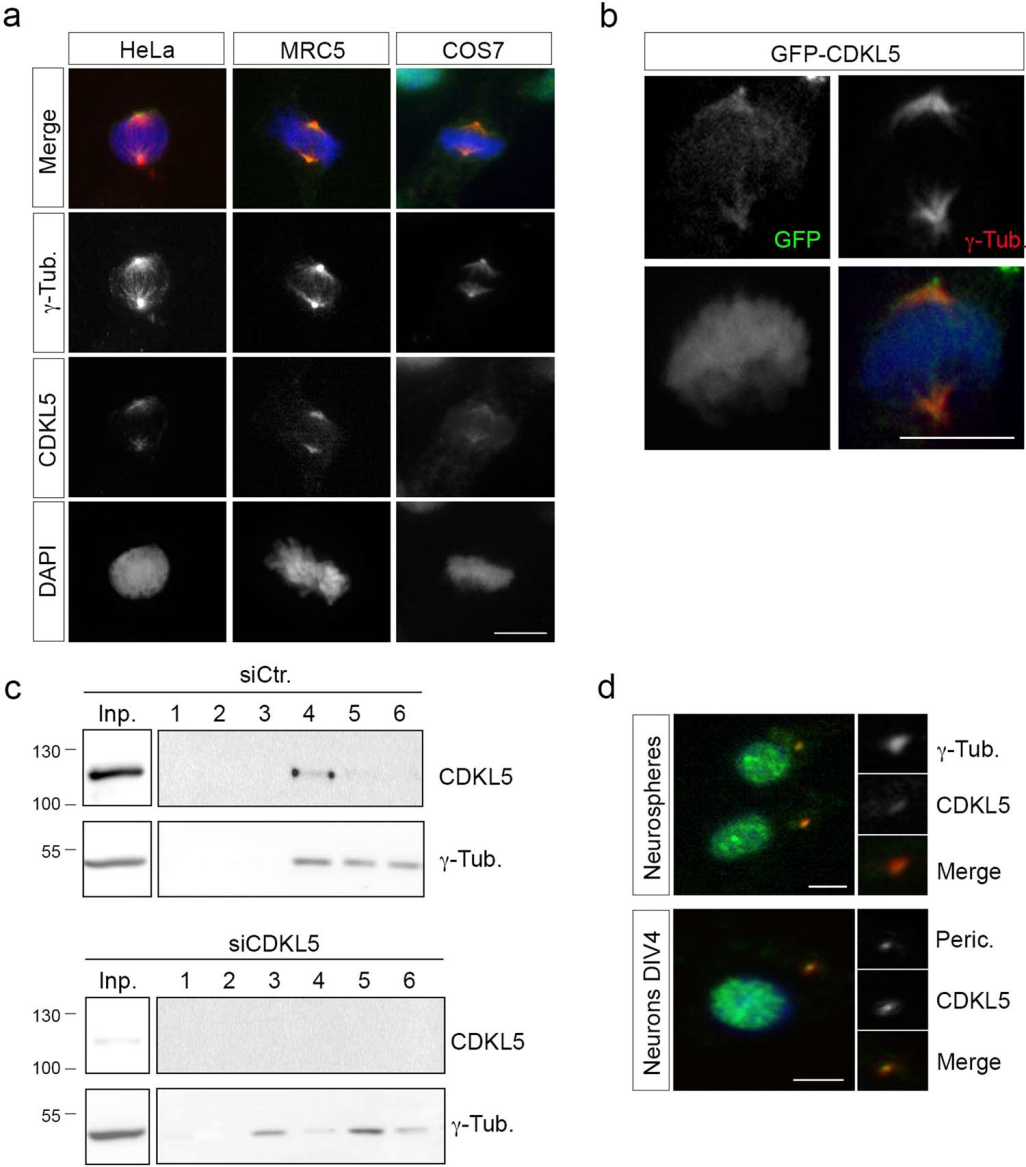
CDKL5 is localized at the centrosome. (**a**) The indicated cells were stained as in Fig. 1. (**b**) Exogenously expressed GFP-CDKL5 (green) localizes at the γ-tubulin positive (red) centrosome in HeLa cells. DAPI staining (blue) was used to visualize DNA. (**c**) HeLa cells were transfected with siCDKL5#1 or a control siRNA (siCtr.); centrosomes were purified 60 h post-transfection and the obtained fractions analyzed by WB with Abs against CDKL5 and γ-tubulin. Input corresponds to approximately 0.6% of the whole cell extract before fractionation. Fractions 1 and 6 are the bottom and top ones, respectively (n = 3 independent experiments). Input and fractions 1–6 are shown as different exposures of the same membrane; full-length blots are presented in Supplementary Figure S7a. (**d**) Neurospheres (upper) and primary hippocampal neurons (DIV4; lower) were stained with Abs against CDKL5 (polyclonal, green) and either γ-tubulin or pericentrin (both red) and DAPI (blue). The panels to the right show the magnified centrosome. Scale bar, 10 µm.

Regarding the centrosomal localization, we confirmed the overlapping staining of CDKL5 and γ-tubulin in different cells, such as SV40-transformed COS7 cells and human fibroblasts, MRC-5 (Fig. 2a). In the latter cells, the centrosomal localization of CDKL5 could be detected also in interphase by using two different antibodies (Abs) (Supplementary Fig. S1a,b). The specificity of the result was further confirmed through siRNA mediated silencing of CDKL5, which led to a significant reduction of the centrosomal CDKL5 staining (Supplementary Fig. S1b,c). Similar to endogenous CDKL5, exogenous green fluorescent protein (GFP)-tagged CDKL5 can be detected at the centrosome in metaphase cells (Fig. 2b). Finally, the presence of CDKL5 at the centrosome was verified by biochemical fractionation of centrosomal proteins obtained from nocodazole/cytochalasin B-treated HeLa cells transfected with a CDKL5 specific siRNA (siCDKL5) or the relative control (siCtr). Western blot (WB) analyses show the presence of endogenous CDKL5 in the γ-tubulin positive centrosomal fractions from siCtr cells but not from siCDKL5 cells (Fig. 2c). Considering the importance of the centrosome for neuronal functions, we also analyzed whether CDKL5 is present at the centrosome in cultured neuronal progenitors and post-mitotic primary hippocampal neurons. Interestingly, CDKL5 is detectable at the centrosome in both cell types (Fig. 2d).

**Figure 3:**
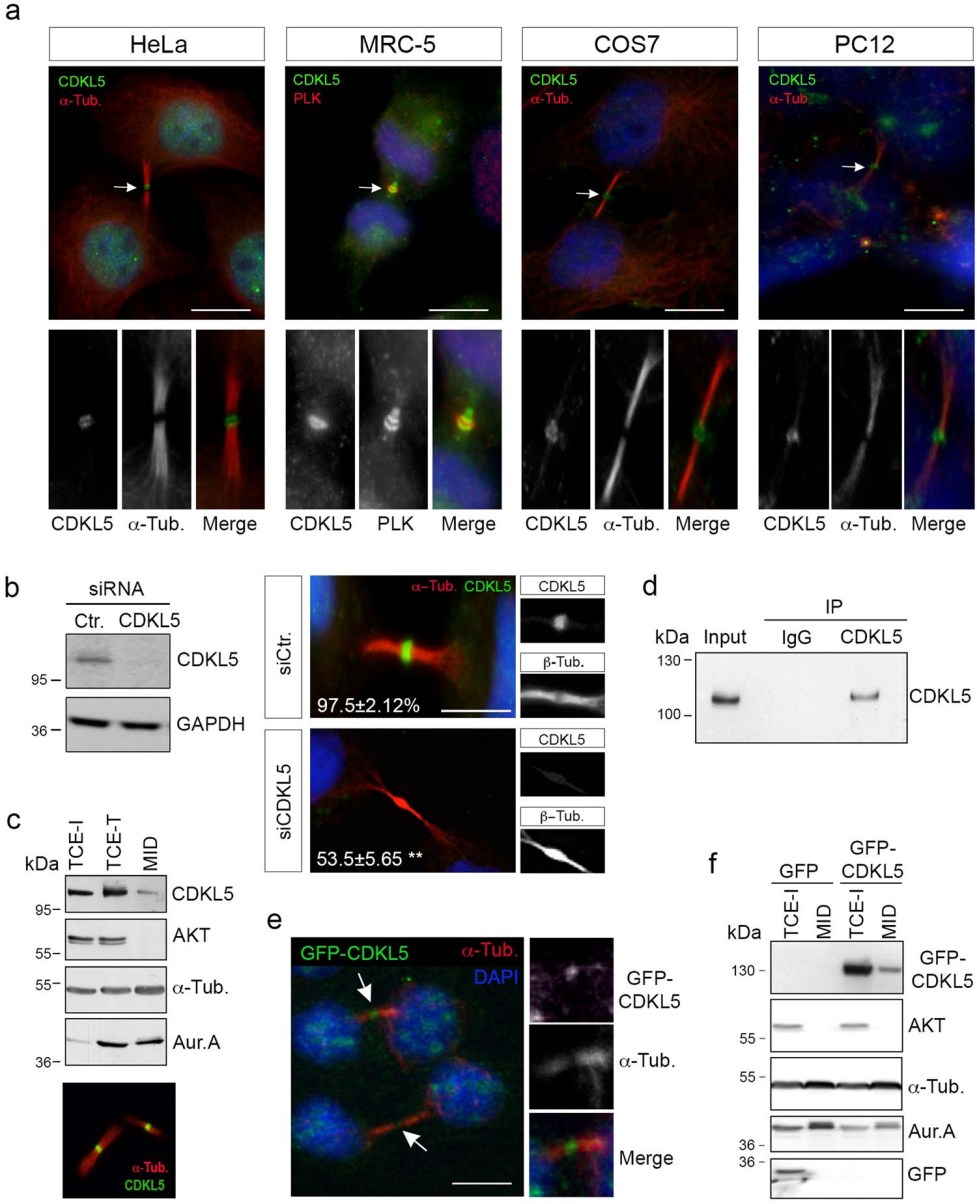
CDKL5 localizes at the midbody. (**a**) The indicated cells were immunostained with Abs against CDKL5 (polyclonal, green) and α-tubulin or PLK-1 (red) to mark the midbody. DAPI staining (blue) was used to visualize DNA. Arrows indicate the CDKL5 positive midbodies, shown enlarged in the lower panels. (**b**) HeLa cells were treated as in Fig. 2c and silencing of CDKL5 expression was verified by western blotting 60 h post siRNA transfection (left). Immunofluorescence analysis (right) showed that only 53.5% ± 5.65 of siCDKL5-treated cells were positive for staining with CDKL5 Ab (polyclonal) at the midbody as opposed to 97.5% ± 2.12 of siCtr cells (n = 2; **p < 0.01; unpaired t-test; 160 midbodies analyzed per condition). Scale bar, 5 µm. (**c**) Total cell extracts of interphase (TCE-I) and telophase (TCE-T) cells were analyzed by WB together with extracts of purified midbodies (MID) with the indicated Abs. Midbody isolation was confirmed by immunofluorescence with Abs against CDKL5 and α-tubulin before extraction (lower panel), (n = 3). (**d**) WB showing immunoprecipitation of a midbody protein extract with polyclonal anti-CDKL5 or anti-IgG. Input corresponds to 10% of the midbody extract before the immunoprecipitation. (n = 2). (**e**) Exogenously expressed GFP-CDKL5 (green) could be detected at the midbody in transfected Hela cells. Arrows indicate GFP-CDKL5 at the midbody. Right panels show the magnified midbody. (**f**) Midbodies (MID) were purified from HeLa cells expressing GFP or GFP-CDKL5 and the extracted proteins were analyzed by WB with the indicated Abs together with an interphase total cell extract (TCE-I) (n = 2). Scale bars in **a** and **e**, 10 µm.

To validate the midbody localization, we first performed IF to confirm the co-staining of CDKL5 with two midbody markers, α-tubulin and PLK-1^22^ in different cell types using two different anti-CDKL5 Abs (Fig. 3a and Supplementary Figure S2). As above, the specificity of the Ab was tested by silencing CDKL5 expression in HeLa cells (Fig. 3b). As biochemical approach, we isolated and purified midbodies from proliferating HeLa cells enriched in telophase by nocodazole treatment, mitotic shake off, and release after nocodazole washout^21^. Successful midbody isolation was assessed by IF (Fig. 3c, lower panel). Midbody extracts (MID) were analyzed by WB and compared with total cell extracts (TCE) from the same number of cells in interphase (TCE-I) and telophase (TCE-T). WB for the mitotic kinases Aurora A and cytoplasmic AKT was used to validate the fractionation protocol. In line with the results obtained by IF, endogenous CDKL5 can be detected in the midbody extracts both by WB (Fig. 3c) and by immunoprecipitation (Fig. 3d). Finally, similar to endogenous CDKL5, exogenous GFP-CDKL5 is detectable at the midbody by IF (Fig. 3e) and in midbody extracts by WB (Fig. 3f).

Altogether, these results show that, beside its well-defined nuclear and cytoplasmic localization, CDKL5 also localizes at the centrosome and midbody in different cell types.

### CDKL5 depletion triggers mitotic spindle multipolarity by centrosome accumulation

**Figure 4:**
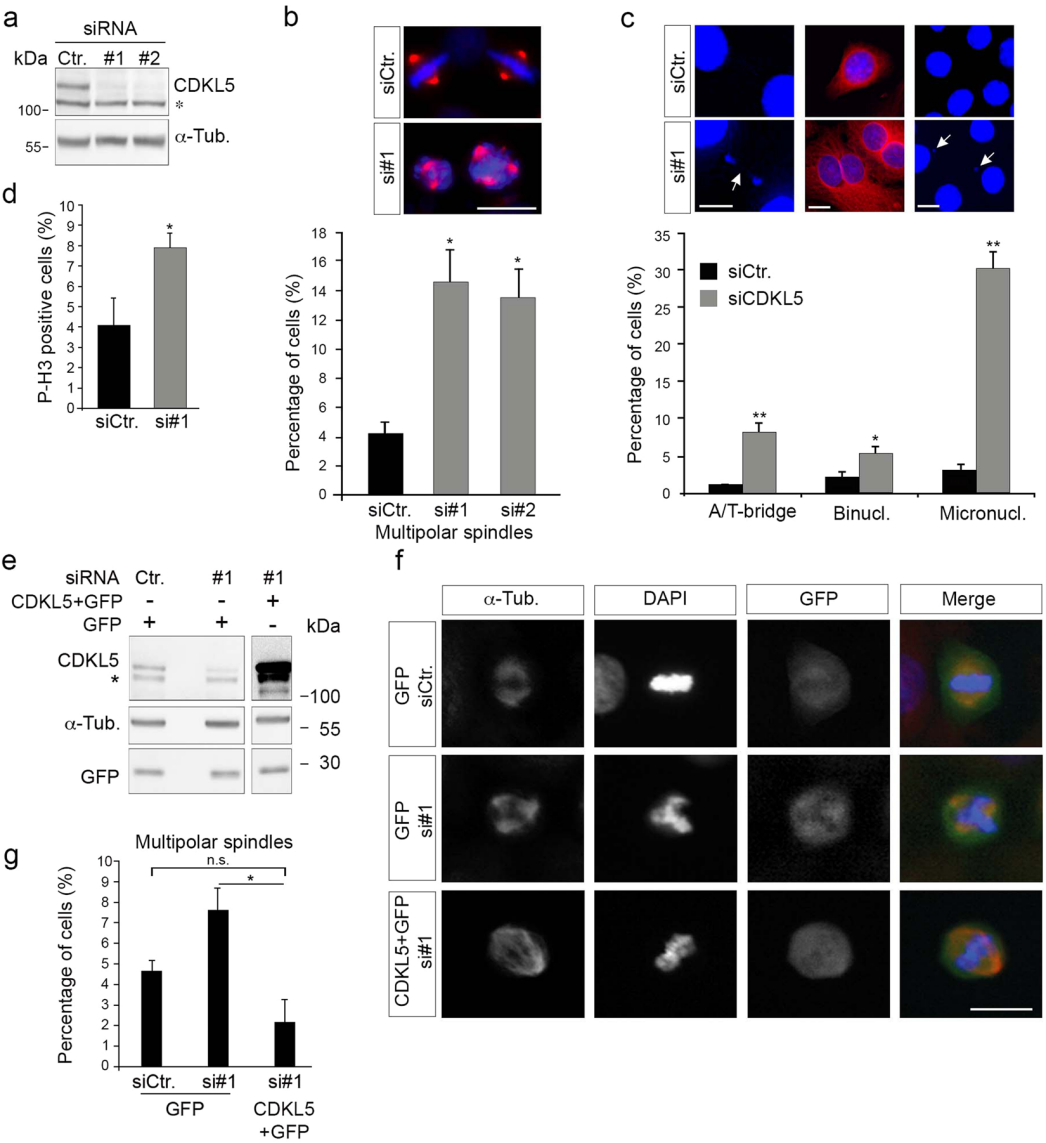
Silencing of CDKL5 is associated with multipolar spindle formation and chromosome segregation defects. (**a**) CDKL5 expression in HeLa cells 60 h after transfection with two different siRNAs targeting CDKL5 or a control siRNA. α-tubulin was used as loading control. The asterisk indicates an unspecific band (**b**,**c**) HeLa cells treated as in **a** were stained against α-tubulin and DAPI 60 h post-transfection. In **b**, the percentage of mitotic multipolar spindles is reported as mean ± S.E.M. (n = 3; *p < 0.05, ANOVA followed by Dunnet’s *post hoc* analysis). In **c**, the frequency of the indicated phenotypes is shown as mean ± S.E.M. (n = 2; *p < 0.05; **p < 0.01; unpaired t-test; approximately 1000 counted cells per condition). In **b** and **c**, representative images of the indicated cells are shown above the graphs with DAPI in blue and α-tubulin in red; scale bar, 10 µm. In **c**, arrows indicate a chromosome bridge and micronuclei. A/T = ana-/telo-phase. (**d**) HeLa cells were treated as in **a** and analyzed for phosphorylated histone H3 (P-H3) 60 h after silencing. The percentage of P-H3 positive cells was calculated in three independent experiments counting approximately 2000 cells (mean ± S.E.M., unpaired t-test). (**e**) CDKL5 was expressed in HeLa cells 60 h post-silencing by transfection of a bicistronic vector expressing also GFP. Vertical cropping was performed to show different exposures of the same membrane; full-length blots are shown in Supplementary Figure S7b. (**f**) Representative images of spindles in GFP-positive cells (green) treated as in **e** by staining against α-tubulin (red) and with DAPI (blue). Scale bar, 10 µm. (**g**) Graph showing number of cells with multipolar spindles upon CDKL5 re-expression in silenced cells. ≥ 60 counted cells per condition (n = 3; mean ± S.E.M, *p < 0.05; ANOVA followed by Dunnet’s *post hoc* analysis). n.s. = not statistically significant.

We then evaluated the phenotype of CDKL5-depleted cells during cell division. IF analyses were performed with HeLa cells 60 h after siRNA transfection when CDKL5 expression is strongly reduced (Fig. 4a). Compared with siCtr, siCDKL5 HeLa cells showed a significant increase in the number of cells with multipolar spindles (Fig. 4b) associated with different chromosome segregation defects including anaphase/telophase chromosome bridges, micronucleation, and binucleated cells (Fig. 4c). In addition, a significant increase in the number of cells positive for the mitotic marker phosphorylated histone H3-S10 (Fig. 4d) supported that an increased number of cells accumulate in mitosis. Similar defects were observed also in siCDKL5 MRC-5 cells (Supplementary Figure S3). To exclude that the observed phenotypes were due to off-target effects, we performed rescue experiments in which siRNA-resistant CDKL5 was co-expressed with GFP from a bicistronic cassette in silenced HeLa cells. As shown in Fig. 4e–g, cells re-expressing CDKL5 have a percentage of multipolar spindles similar to siCtr cells expressing only GFP.

Spindle multipolarity can be due to defects in microtubule (MT) dynamics or to numeral and structural centrosome alterations^23^. To assess the underlying cause of multipolarity in CDKL5 depleted cells, we first evaluated whether silencing of CDKL5 impairs MT dynamics in COS7 and MRC-5 cells by analyzing the regrowth of MTs after their nocodazole mediated disruption. No significant difference was observed in the aster size of newly formed MTs between siCtr and siCDKL5 cells, suggesting that CDKL5 depletion does not alter the microtubule nucleation activity of these cells (Supplementary Figure S4).

**Figure 5:**
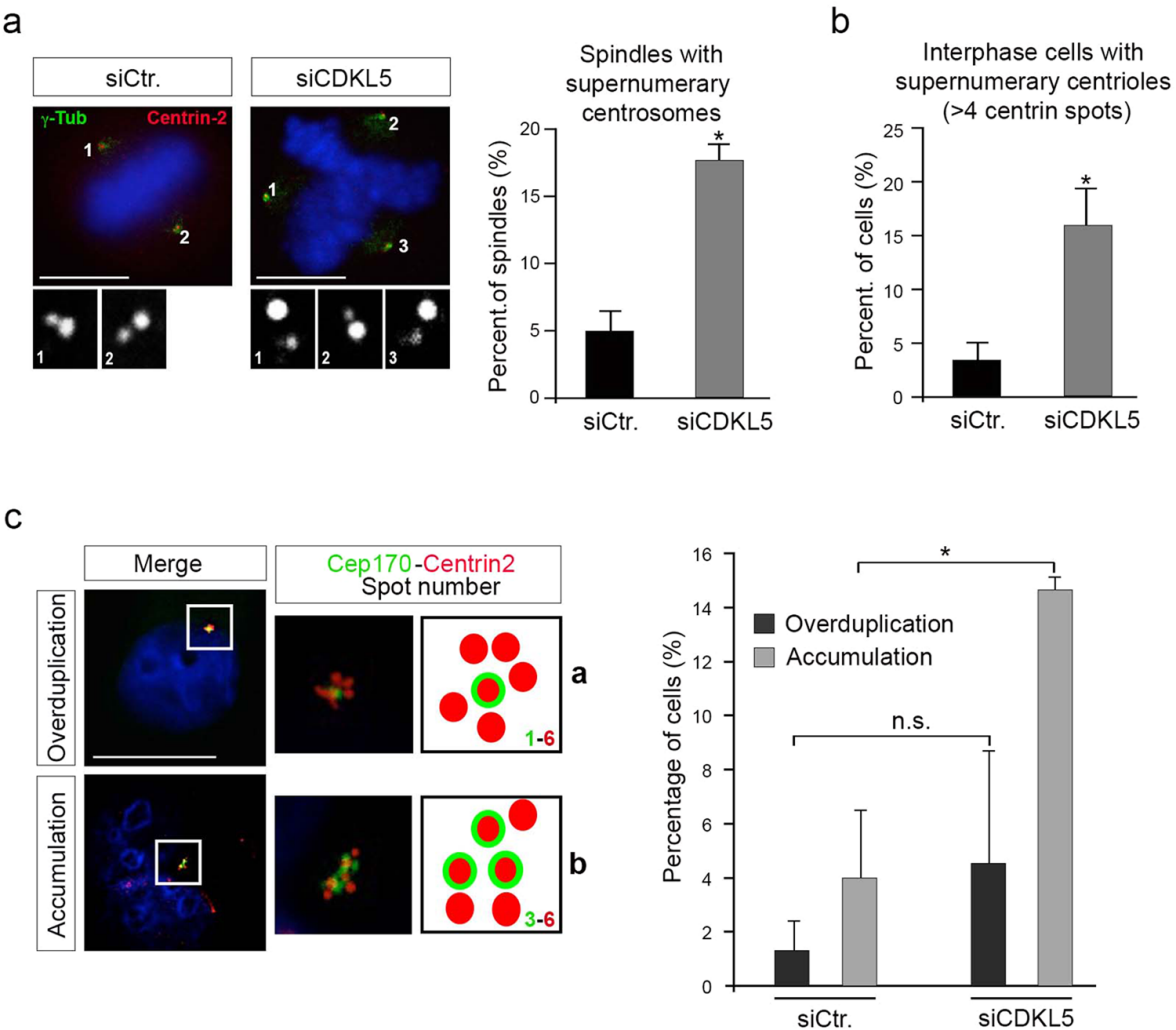
Spindle multipolarity in CDKL5 depleted cells is due to centrosome accumulation. (**a**–**c**) HeLa cells were silenced as in Fig. 4a and analyzed by IF 60 h after transfection. In (**a**) cells were stained with Abs anti-γ-tubulin and anti-centrin-2 to mark the spindle poles and the centrioles, respectively. DAPI staining (blue) was used to visualize DNA. The insets below show the magnified centrin-2 signal in the correspondingly numbered poles. The percentage of spindles with supernumerary centrosomes is reported to the right as mean ± S.E.M. (n = 2; *p < 0.05; unpaired t-test; approximately 70 spindles were analyzed per condition). In (**b**) cells were immunostained with centrin-2 and the percentage of interphase cells with more than 4 centrioles was reported as mean ± S.E.M. (n = 3; *p < 0.05; unpaired t-test; approximately 100 cells were analyzed per condition). In **c** cells were stained with anti-cep170 (green) and anti-centrin-2 (red) Abs. DAPI staining (blue) was used to visualize DNA. Representative images of different siCDKL5 phenotypes are shown on the left. The percentage of cells with the indicated phenotypes is reported to the right as mean ± S.E.M (n = 2; *p < 0.05; unpaired t-test; approximately 200 cells were analyzed per condition). n.s. = not significant. Scale bar, 10 µm.

We proceeded evaluating whether CDKL5 depletion might lead to loss of centrosome integrity. Microscopy analyses of siCDKL5 HeLa cells revealed a large number of mitotic spindles with more than two centrosomes, that appear grossly normal, as shown by the staining with the centriolar marker centrin-2 (*i*.*e*., each centrosome presents two spots of centrin-2; Fig. 5a and Supplementary Figure S5). Notably, we never observed either one centrin-2 dot per spindle pole or a centrin-2 negative spindle pole (*i*.*e*., zero out of 140 spindles); we thus excluded loss of centriole cohesion or centrosome fragmentation as causative of the multipolar spindles. Furthermore, a significant increase of interphase cells with supernumerary centrioles (more than four per cell) was detected in siCDKL5 compared to siCtr cells (Fig. 5b). Altogether, these data support the hypothesis that spindle multipolarity might be due to supernumerary centrosomes rather than to the loss of structural centrosome integrity.

Supernumerary centrosomes can arise through centriole overduplication or accumulation^24^. To distinguish between these two events, double IF analysis for centrin-2 and Cep170 was performed^25, 26^. Cep170, a marker for mature, maternal centrioles, can be used to assess the ratio of mature, maternal centrioles and immature, daughter centrioles. This ratio is balanced during centrosome accumulation whilst centrosome duplication causes an excessive number of daughter centrioles compared to maternal ones. Quantification of the Cep170 and centrin-2 staining revealed that CDKL5-deficient cells show a significant increase in centriole accumulation compared to siCtr cells whereas no significant difference was observed in centriole overduplication (Fig. 5c).

Taken together, these results indicate that CDKL5 depletion triggers mitotic spindle multipolarity through centrosome accumulation.

### CDKL5 depleted cells show polyploidization and cytokinesis failure

**Figure 6:**
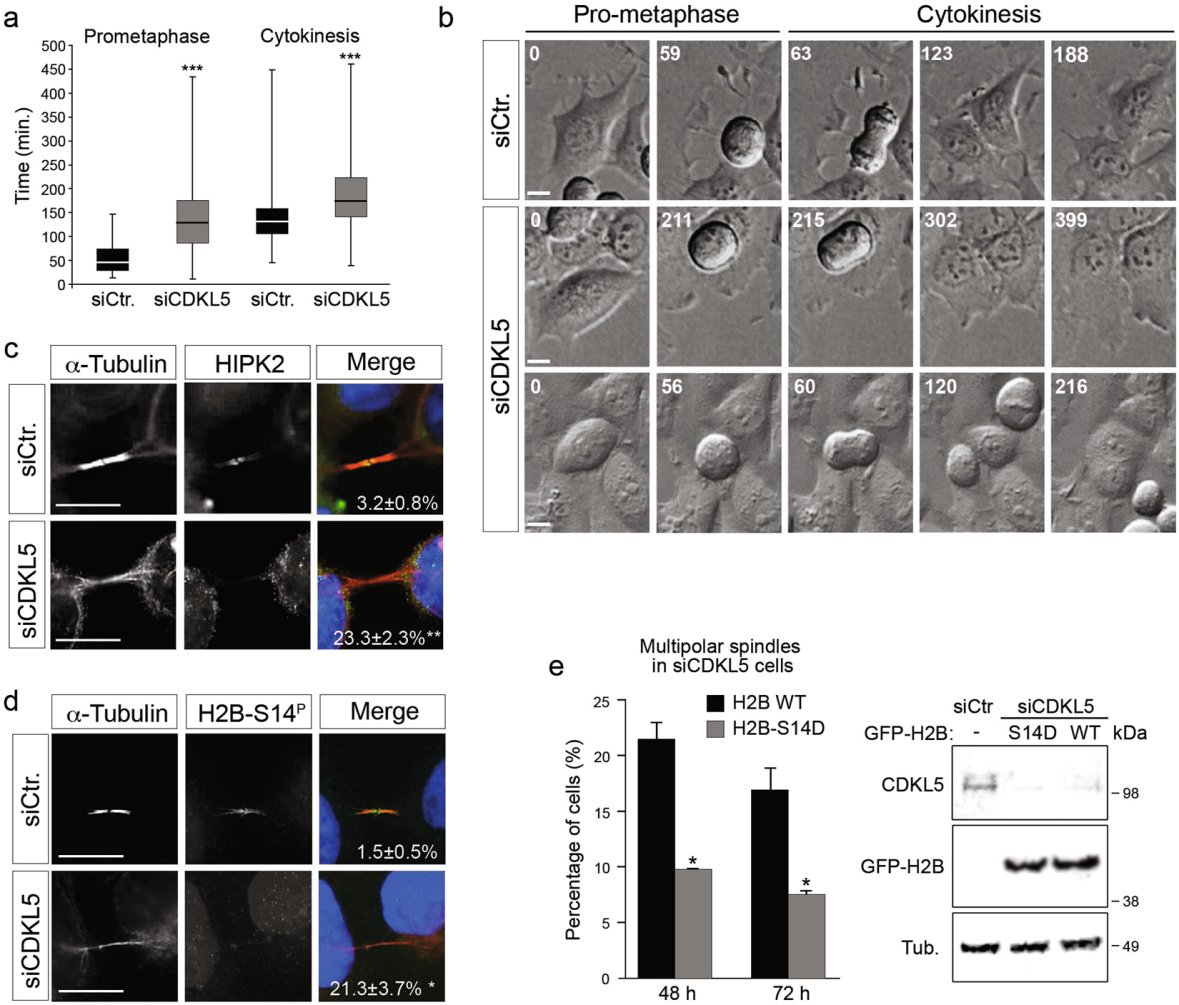
CDKL5 depletion is associated with cytokinesis failure and impaired HIPK2/H2B activity. (**a**) Indicated HeLa cells were analyzed 60 h post-silencing by time-lapse videomicroscopy. The duration of prometaphase and cytokinesis is reported in box plot graphs (***p < 0.001, unpaired *t* test). (**b**) Still images related to Supplementary Movies S1, S2 and S3, respectively upper, middle and lower panels. Representative still images of siCDKL5 cells with very long cytokinesis time (middle panels) and of siCDKL5 cells that fail cytokinesis and form binucleated cell (lower panels) are showed. Time is indicated in minutes. Scale bar, 10 µm. (**c**, **d**) Midbody localization of HIPK2 (**c**) and H2B phosphorylated at S14 (**d**; H2B-S14^P^) was analyzed in the indicated HeLa cells 60 h post-silencing by staining with Abs against α–tubulin (red) and HIPK2/H2B-S14^P^ (green) and with DAPI (blue). The percentage of cells with HIPK2/ H2B-S14^P^ negative midbodies is indicated for each condition (n = 2; mean ± S.E.M., *p < 0.05; **p < 0.01 unpaired t-test; approximately 80 midbodies were analyzed per condition). Scale bar, 10 µm. (**e**) Graph showing percentage of multipolar spindles in siCDKL5 cells expressing GFP-H2B WT or GFP-H2B-S14D 48 and 72 h after siRNA transfection (n = 2; *p < 0.05; unpaired t-test; approximately 80 cells were analyzed per condition). WB showing CDKL5 and GFP-H2B protein levels 72 h after siRNA transfection is reported.

Centrosome accumulation can originate via different pathways, including endoreduplication, mitotic slippage (mitosis without chromosome segregation), cytokinesis failure, and cell fusion^24^. We thus analyzed siCDKL5 cells by *in vivo* time-lapse imaging. Compared to controls, siCDKL5 cells show a significantly longer prometaphase time [from the round-up to chromosome segregation; t = 148.3 ± 91.34 min in siCDKL5 (n = 74) *versus* 59.07 ± 33.64 min in siCtr (n = 73); p < 0.001]. In these cells, we also observed a significantly longer cytokinesis time [from the cleavage furrow ingression to cell daughter separation; t = mean ± Standard deviation = 186.38 ± 78.39 min in siCDKL5 (n = 66) *versus* 139.81 ± 62.81 min in siCtr (n = 62); p < 0.001] and cytokinesis failure resulting in the formation of binucleated cells (Fig. 6a,b and Supplementary Movies S1, S2 and S3). We did not observe any sign of cell death or mitotic slippage (*i*.*e*. no cells that, after round-up, re-adhere without chromosome segregation) in either siCtr or siCDKL5 cells during the imaging session (Fig. 6a,b, Supplementary Movies S1 and S2 data not shown).

Altogether, these findings show that CDKL5 depletion leads to polyploidization and cytokinesis failure.

### CDKL5 depletion impairs HIPK2/H2B midbody activity

The midbody localization of CDKL5, the longer cytokinesis time, and the increment of binucleated cells upon silencing of CDKL5 (Figs 4c and 6a,b and Supplementary Figure S3c) support the hypothesis that depletion of the kinase might affect cytokinesis. Thus, to get insight into the molecular mechanism involved in the observed cytokinesis failure, we evaluated whether CDKL5-depleted cells show any midbody defects. The localization of different structural and functional midbody factors including kinases (*i*.*e*., Aurora B, PLK1, HIPK2) and microtubule-associated proteins (*i*.*e*., MKLP1, MgcRacGap1, PRC1, and Spastin)^21, 22, 27, 28^ were assessed by IF in siCtr and siCDKL5 HeLa cells. Among the analyzed factors, we found only HIPK2 to be absent from the midbody in a high percentage of siCDKL5 cells compared with the siCtr cells (Fig. 6c and Supplementary Figure S6). HIPK2 contributes to cytokinesis through the phosphorylation of extrachromosomal histone H2B at S14 at the midbody^21^. Thus, using a phospho-specific Ab we analyzed the presence of phosphorylated H2B-S14 at the midbody in cells silenced for CDKL5. Consistently with the delocalization of HIPK2, we did not detect any phosphorylation of H2B-S14 at the midbody in a high percentage of CDKL5-depleted cells (Fig. 6d) suggesting that CDKL5 contributes to cytokinesis by regulating HIPK2/H2B activity at the midbody.

The most evident phenotype we have observed in the CDKL5 depleted cells is spindle multipolarity, which we linked to polyploidization and centrosome accumulation. Moreover, we observed a cytokinesis failure that is known to depend on HIPK2/H2B activities at the midbody. Thus, we hypothesized that loss of HIPK2 and H2B-S14 phosphorylation at the midbody might trigger a cascade of events starting from cytokinesis failure leading to centrosome accumulation and the formation of multipolar spindles. We thus asked whether spindle multipolarity in siCDKL5 HeLa cells could be rescued by the expression of a phosphomimetic H2B-S14D derivative, which was previously shown to be capable of overcoming cytokinesis failure in HIPK2-defective cells^21^. As shown in Fig. 6e, the expression of H2B-S14D significantly reduced the number of cells with multipolar spindles in siCDKL5 cells indicating that CDKL5 contributes to cytokinesis through HIPK2-mediated H2B phosphorylation.

## Discussion

In the last ten years genetic lesions in *CDKL5* have been found in patients with neurologic disorders characterized by the early onset of often intractable seizures, intellectual disability, and impaired motor control^2^. Given the huge repercussion of *CDKL5* mutations on brain functions, most studies have so far focused on the role of this kinase in neurons and very few pieces of information are yet available regarding its role in proliferating cells. Few data have suggested that CDKL5 is involved in cell proliferation^11, 12^, but the subcellular localization and the function of this kinase during the cell cycle are still missing.

Here we present evidence that CDKL5 contributes to faithful cell division. Indeed, we found that the acute ablation of CDKL5 leads to multipolar spindle formation and cytokinesis failure in HeLa and in MRC-5 cells. The increase in the duration of prometaphase and the presence of micronucleated cells suggest that chromosome segregation occurs with reduced fidelity in these cells and that genome instability might be linked to CDKL5 dysfunctions. Therefore, it might be relevant to investigate further genome stability in *Cdkl5*-null or defective backgrounds.

In accordance with a role of CDKL5 in cell division, we observed that its subcellular localization is highly dynamic throughout mitosis and cytokinesis. Indeed, we found an accumulation of CDKL5 at the centrosome from prophase to anaphase and at the midbody in cytokinesis. Among the various midbody factors that we analyzed, only HIPK2 was absent from the midbody in CDKL5 depleted cells. The concomitant reduction of phosphorylated H2B at the midbody and the capacity of the phospho-mimetic H2B-S14D to rescue multipolarity in CDKL5 deficient cells suggest a causative link between the midbody defects and the aberrant mitotic spindles. This is reinforced by the increased number of centrosomes that appear to arise through accumulation rather than through centrosome duplication defects and by the observation that multipolarity appears to be strictly associated with polyploidy after CDKL5 depletion in the diploid MRC-5 cells (Supplementary Figure S3d and e). Even if further studies are warranted to explore the precise mechanism through which CDKL5 depletion leads to the midbody deprivation of HIPK2, our results highlight a novel function of CDKL5 in cytokinesis through the regulation of HIPK2/H2B at the midbody.

At this point, we cannot ruled out that CDKL5 has additional roles in early mitotic stages (prometaphase and metaphase). Indeed, the longer prometaphase time (Fig. 6a,b) and the higher frequency of chromosome bridges and micronuclei compared to the reduced number of binucleated cells (Fig. 4c) that we observed in siCDKL5 cells suggest that CDKL5 depletion might also induce defects in chromosome alignment and/or segregation. Such defects cannot only be linked to midbody alterations.

Conversely, we do not observe any apparent structural centrosome defects in CDKL5 deficient cells. However, this does not preclude the existence of centrosome-associated CDKL5 functions that may emerge from further studies. Interestingly, it is to note that the large group of proteins that have been functionally linked to the centrosome includes MeCP2^29^, the main cause of Rett syndrome and an interactor and *in vitro* substrate of CDKL5^30, 31^. Whether a cross-talk between CDKL5 and MeCP2 exists at the centrosome still remains to be understood. Of note, the reduced MT nucleation capacity of centrosomes in MeCP2 deficient cells, but not in cells silenced for CDKL5, indicate that the two proteins have at least some separate functions in this organelle^29^.

Previous loss- and gain-of-function studies in SH-SY5Y neuroblastoma cells suggested that CDKL5 inhibits cell proliferation^11^. In agreement, in the developing mouse brain, genetic inactivation of *Cdkl5* increases BrdU and Ki67 positivity^12^. Even if we observed an increased positivity for phosphorylated H3-S10 in our CDKL5-depleted cells, we did not find any increase in cell proliferation (data not shown), suggesting that the phospho-H3 positivity might be due to a prolonged cell division time. Our results, showing a role of CDKL5 in cell division, suggest that the influence of CDKL5 on cell proliferation might be linked to its effects on genome stability. This would be in agreement with the observation made in neuroblastoma patients, in which high expression of CDKL5 correlates with an increased overall survival^11^. However, the action of CDKL5 in tumorigenicity is definitively more complex since, at variance with neuroblastomas, in glioblastomas, CDKL5 was shown to be required for tumor cell survival and increased CDKL5 expression was found to be associated with reduced overall survival^32^. Future studies will be essential to investigate the role of CDKL5 in tumorigenesis.

Although defects in CDKL5 have mainly been associated with a neurological disease we consider likely that its influence on cell cycle progression may be of relevance also in the pathology. In fact, it is well known that the precise control of the cell cycle critically regulates neurogenesis, neuronal differentiation and migration^33^ and defects in neuronal progenitor proliferation and differentiation seem to be a convergence point in many neurodevelopmental disorders^34^. Of interest, defects in centrosomal proteins are known to impinge on neuronal migration and axon formation^35^. We thus speculate that the defects in radial neuronal migration and axon specification that have previously been associated with CDKL5 knock-down may be linked to a function of CDKL5 at this organelle^6, 9^. Altogether, a further understanding of the precise role of CDKL5 in cell cycle progression and at the centrosome might be relevant to recognize the origin of some features of the human pathology associated with CDKL5.

## Materials and Methods

### Ethics Statement

Protocols and use of animals were approved by the Animal Ethics Committee of the University of Insubria and in accordance with the guidelines released by the Italian Ministry of Health. Adult mice were euthanized by cervical dislocation.

### Plasmids

pGFP-CDKL5, expressing the 107 kDa hCDKL5 isoform has been described elsewhere^36^. pCAGGS-CDKL5-ires-GFP, expressing a siRNA resistant form of CDKL5, was cloned by inserting an EcoRI-EcoRV digested PCR product containing the murine CDKL5 cDNA (NP_0010795) into pCAGGS-ires-GFP digested with EcoRI and SmaI. siRNA resistant CDKL5 contains three nucleotide changes in the third position of codons 25–27 (ggagttgra =  > ggcggagtt). pBosH2B-GFP and pBosH2BS14D-GFP have been described in Rinaldo *et al*.^21^.

### Antibodies

Antibodies (Abs) against the following proteins were used: β-actin (Sigma-Aldrich, St Louis, MO, USA, A5441), α-tubulin (Sigma-Aldrich, T6074), β-tubulin (Sigma-Aldrich, C4585), γ-tubulin (Sigma-Aldrich, T5326), CDKL5 (polyclonal, Sigma-Aldrich, HPA002847; monoclonal Santa Cruz, clone D-12, sc-376314), AKT (Cell Signaling, 4685), Aurora kinase A (Sigma-Aldrich, A1231, clone 35C1), GFP (Roche Diagnostics Ltd, 1814460), PLK-1 (Santa Cruz, sc-17783), phospho-H3-S10 (Abcam, ab14955), H2SB14-P (Cell Signaling, 6959); centrin-2 (Santa Cruz, sc-27793), Cep170 (ThermoFisher Scientific, 41-3200) and HIPK2 (rabbit polyclonal^21^); MgcRacGAP1 (Abcam, ab2270), MKLP1 (Santa Cruz; sc-22793); PRC1 (Santa Cruz, Sc-8356); spastin (Santa Cruz, Sc-53443), CREST centromere protein (Antibodies Inc., 15-234-0001). HRP-conjugated goat anti-mouse or anti-rabbit secondary Abs for immunoblotting were purchased from Thermo Fisher Scientific. DAPI and secondary Alexa Fluor anti-rabbit, anti-mouse, anti-human, and anti-goat Abs for immunofluorescence were obtained from Life Technologies while FITC- or TRITC-conjugated Abs were from Jackson Immuno Research Lab.

### Cell cultures, transfections and RNA interference

Human cervix adenocarcinoma HeLa cells, the monkey SV-40-transformed kidney COS7 cells, and human fetal lung fibroblasts MRC-5 cells were maintained in DMEM (Dulbecco’s modified Eagle’s medium; Sigma-Aldrich) supplemented with 10% FBS, L-glutamine, 100 units/ml penicillin, and 100 μg/ml streptomycin at 37 °C with 5% CO_2_. Rat adrenal gland pherochromocytoma PC12 cells were maintained in RPMI (Gibco, Thermo Fisher Scientific) supplemented with FBS, L-glutamine, penicillin, and streptomycin as above; these cells were seeded on poly-D-lysine coated coverslips.

For transfection, cells were cultured in 6- or 12-well dishes and transfected with plasmids using Lipofectamine^TM^ 2000 (Life Technologies) following the manufacturer’s protocol. Cells were collected or fixed 24 h post-transfection. For siRNA transfection, 20 nM siRNA oligonucleotides targeting CDKL5 or control GL2 siRNA targeting the luciferase gene (shCDKL5#1: CUAUGGAGUUGUACUUAAAUU; shCDKL5#2: GCAGAGUCGGCACAGCUAUUU; siCtr. 5′CGUACGCGGAAUACUUCGAUU3′) were transfected into HeLa or MRC-5 cells using Lipofectamine^TM^ RNAiMAX (Life Technologies).

For CDKL5 rescue experiments, a siRNA resistant pCAGGS-CDKL5-ires-GFP vector was transfected 60 h after CDKL5 silencing and cells analyzed after another 24 h. For H2B-GFP or H2B-S14D expression, encoding vectors were transfected 24 h after CDKL5 silencing and cells were analyzed 24 and 48 h post-transfection.

### Primary neuronal cultures

Primary hippocampal cultures were prepared from embryonic day 17 (E17) CD1 mouse embryos, considering the day of the vaginal plug as E0, as described previously^7^ and plated on poly-L-lysine coated plates (densities: 2,5 × 10^3^/cm^2^).

### Neurosphere preparation

Neurospheres were prepared as previously described^37^. Briefly, E15.5 embryos were individually dissected in PBS and the neocortex was transferred in Dulbecco’s Modified Eagle Medium/F12 (DMEM) and dissociated by extensive enzymatic digestion with Papaine (Sigma-Aldrich). Cells were grown in medium containing DMEM/F12, 0.66% glucose, L-glutamine 1%, Pen/Strep 1%, 4 µg/mL of heparin, hormone mix with the addition of either 20 ng/mL epidermal growth factor (EGF) and 10 ng/mL basic fibroblast growth factor (bFGF). In such conditions, cells spontaneously formed neurospheres. Neurospheres were then dissociated into single-cell cultures and plated on matrigel-coated coverslips (BD Bioscience).

### Centrosomal fractionation

Centrosome fractionation was performed as previously described^29^. Briefly, exponentially growing cells were treated with 10 μg/ml nocodazole and 5 μg/ml cytochalasin B (both from Sigma-Aldrich) for 90 min, followed by hypotonic lysis. Centrosomes were harvested by centrifugation onto a 20% Ficoll cushion and further purified by centrifugation through a discontinuous (70%, 50% and 40%) sucrose gradient. Fractions of 0.3 ml were collected and analyzed by WB.

### Western blotting (WB) and immunoprecipitation (IP)

Cells were lysed with lysis buffer (50 mM Tris-HCl pH 7.5, 150 mM NaCl, 1% Triton X-100, 1 mM EDTA, 1 mM DTT) with addition of protease inhibitor cocktail (Sigma-Aldrich) and PhosSTOP (Roche Diagnostics). After 30 min on ice, the lysates were clarified by centrifugation and the supernatants collected. Samples were separated by either 10% or 8% SDS-PAGE, transferred to nitrocellulose membranes, and blocked in 5% non-fat milk in TBS (20 mM Tris-HCl, pH 7.5, 150 mM NaCl) with 0.2% Tween-20 (T-TBS). Blots were incubated with primary Abs overnight at 4 °C, washed in T-TBS, and incubated with appropriate secondary Abs for 1 h at room temperature. After extensive washes, blots were developed with either West PICO Chemiluminescence kit (Pierce) or with ECL Prime Western Blotting Detection Reagent (GE Healthcare).

### Immunofluorescence (IF)

Cells were grown on poly-L-lysine coated coverslips and fixed in ice-cold methanol or 2% formaldehyde, washed three times in phosphate buffered saline (PBS), permeabilized in 0.25% Triton X-100 in PBS for 10 min and then blocked in 5% bovine serum albumin (BSA) in PBS for 1 h before the required primary Abs were applied. For the staining of γ-tubulin and centrosome associated proteins, cells were permeabilized before fixation in PHEM (60 mM Pipes, 25 mM Hepes, 10 mM EGTA, and 2 mM MgCl_2_) with 0.5% Triton X-100 for 2 min and then fixed in 4% paraformaldehyde.

### Microscopy on fixed samples

For Figs 1, 2a,b,c, 3a,e and 4b,f and Supplementary Figures S2 and S3a, preparations were examined under a NikonEclipse Ni upright microscope equipped with a 100X (1.35 NA) oil immersion objective and a Nikon DIGITAL SIGHT DS-U1 CCD camera. Images were acquired using the NIS-Elements BR 4.13.04 software. For Supplementary Fig. S1a and S1, microscopy analysis was performed using a Olympus U-RFL-T microscope equipped with a 100X (1.35 NA) oil immersion objective and a Rriga R1 CCD camera (QImaging). Images were acquired using Ocular 1.0 software. For Supplementary Figure S4, microscopy was performed using a confocal laser-scanning microscope (model TCS SP8; Leica) with a 63X NA 1.2 oil immersion objective (Leica). Images were acquired using LAS-AF Lite software. For the Figs 3b, 4c and 6c,d and Supplementary S5, preparations were examined under an Olympus Vanox microscope equipped with a 100X (1.35 NA) oil immersion objective and a Tucsen tch-1.4ice CCD camera (Tucsen photonics co., LTD). Images were acquired using Isocapture 4.1.3 software (Tucsen photonics co., LTD). For Fig. 5a, microscopy was performed using a Nikon Eclipse 90i microscope equipped with a oil immersion Plan Fluor 100x objective (N.A. 1.3; Nikon) and a Qicam Fast 1394 CCD camera (QImaging). Image acquisition, deconvolution and Extended Depth of Focus on z-serial optical sections were performed using Nis-Elements HC 4.2 (Nikon); Images shown were Maximum Intensity Projections from z-stacks.

### Midbody purification

HeLa cells were enriched in telophase by treatment with nocodazole (100 ng/ml) for 4 h followed by mitotic shake-off. Nocodazole was washed out and the collected cells incubated for 80 min to reach telophase. Midbodies were isolated as described by Kuriyama *et al*.^38^ and extracted in extraction buffer (50 mM Tris-HCl pH 7.4, 150 mM NaCl, 0.1% SDS, 0.5% NP40, 5 mM EDTA) supplemented with protease- (Sigma-Aldrich) and phosphatase-inhibitors (Roche, Diagnostiics). Total cell extracts (TCE) from asynchronous and telophase enriched cells were analyzed in parallel.

### Live-cell imaging

Cells were seeded in 8-well slides (80826, ibiTreat) and observed under an Eclipse Ti inverted microscope (Nikon) using a 40x objective (Plan Fluor, N.A. 0.60, DIC, Nikon). During the observation, cells were kept in a microscope stage incubator at 37 °C and 5% CO_2_. DIC images were acquired by using a DS-Qi1Mc camera. Image and video processing were performed with NIS-Elements AR 3.22.

### Statistics

For statistical analysis we assessed the normality distribution of data using the Shapiro-Wilk normality test: student’s *t* test or parametric ANOVA followed by Dunnet’s *post hoc* test was applied to determine the significance of quantitative experiments when the data distribution was normal. In other cases we used non-parametric Kruskal Wallis with Dunn’s *post hoc* test.

## Electronic supplementary material

Movie S1

Movie S2

Movie S3

Supplementary information
