# Supplementary material for: CDKL5 localizes at the centrosome and midbody and is required for faithful cell division
Source: Sci Rep. 2017 Jul 24;7:6228. doi: 10.1038/s41598-017-05875-z (PMC5524905; doi:10.1038/s41598-017-05875-z)
Supplement: Supplementary file 4 — Supplementary information [file 41598_2017_5875_MOESM4_ESM.pdf]

CDKL5 localizes at the centrosome and midbody and is required for faithful cell division

Isabella Barbiero[§1], Davide Valente[§2], Chetan Chandola[§1^], Fiorenza Magi[§3^], Anna Bergo[1], Laura Monteonofrio[2], Marco Tramarin[1], Maria Fazzari[4], Silvia Soddu[2], Nicoletta Landsberger[4], Cinzia Rinaldo[2,3+*], Charlotte Kilstrup-Nielsen[1+*]

[1]Department of Biotechnology and Life Sciences, University of Insubria, 21052 Busto Arsizio, Italy

[2]Institute of Molecular Biology and Pathology (IBPM), National Research Council (CNR), c/o Sapienza University, 00185 Rome, Italy

[3]Unit of Cellular Networks and Molecular Therapeutic Targets, Department of Research, Advanced Diagnostic, and Technological Innovation, Regina Elena National Cancer Institute – IRCCS, 00144 Rome, Italy

[4]Department of Medical Biotechnology and Translational Medicine, University of Milan, 20090 Segrate, Italy

[§]These authors contributed equally to the work

*corresponding author: c.kilstrup-nielsen@uninsubria.it.

[+]co-last authors.

^Present addresses: CC: Centre for Drug Research, Division of Pharmaceutical Biosciences, Univeristy of Helsinki, Finland; FM: Laboratory of Biomedical Research "Fondazione Niccolò Cusano per la Ricerca Medico-Scientifica", Niccolò Cusano University, Rome, Italy.

Correspondence: Dr Cinzia Rinaldo, IBPM-CNR, Laboratory of Genetics, Via degli Apuli, 4, I-00185 Rome, Italy. Phone: +39-06-49917537; e-mail: cinzia.rinaldo@uniroma1.it; cinzia.rinaldo@ifo.gov.it

Dr. Charlotte Kilstrup-Nielsen, University of Insubria, Via Manara 7, 21052 Busto Arsizio, Italy. Phone +39-0331339430; e-mail: c.kilstrup-nielsen@uninsubria.it

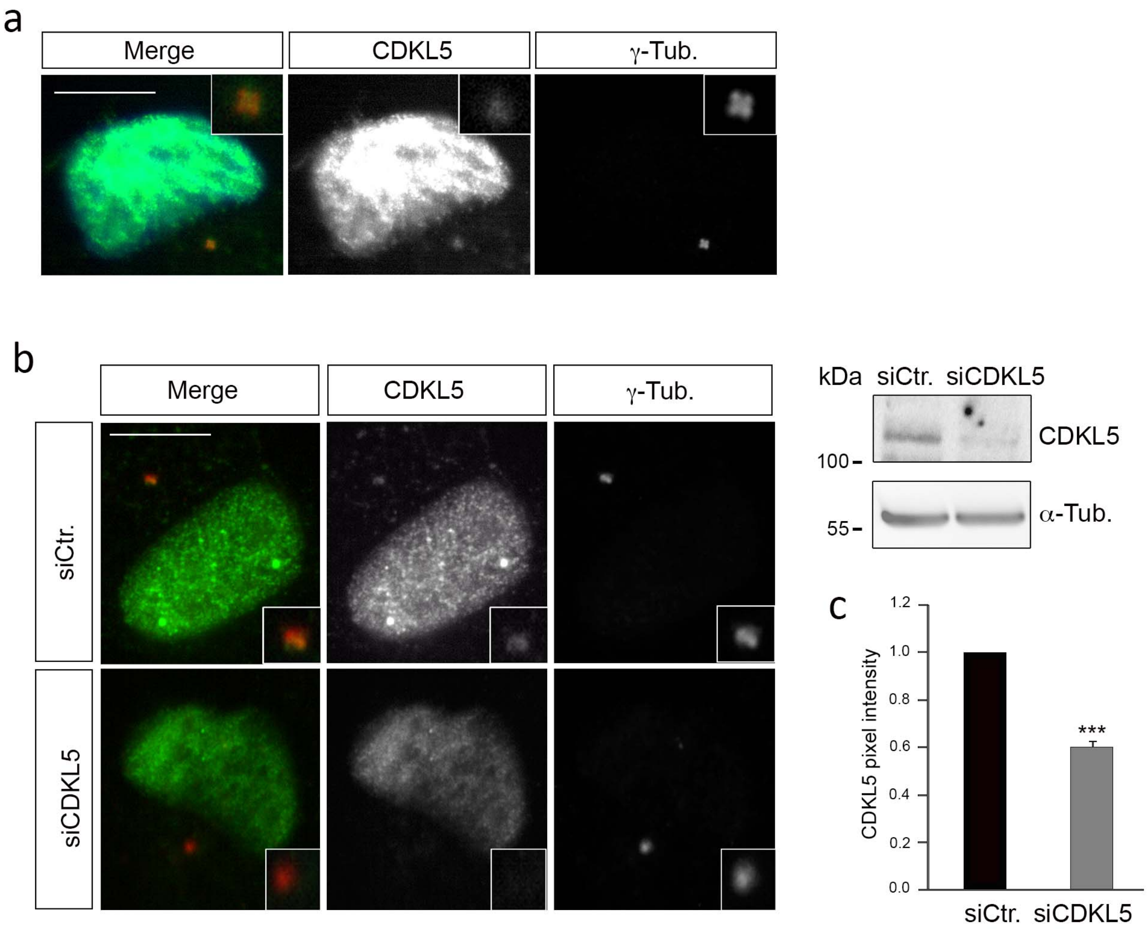

Supplementary Figure S1. Specificity of centrosomal localization of CDKL5. **(a)** Interphase MRC-5 cells were stained with monoclonal anti-CDKL5 Ab (green) and anti-γ-tubulin Ab (red). The inset shows the magnified centrosome. **(b)** MRC-5 cells were transfected with a control siRNA (siCtr.) or a CDKL5-specific siRNA (siCDKL5#1). Three days post-silencing the cells were stained with polyclonal anti-CDKL5 Ab (green) and anti-γ-tubulin Ab (red). The inset shows the magnified centrosome. Silencing efficiency was verified by western blotting using α-tubulin as loading control. **(c)** The fluorescence intensity of CDKL5 at the centrosome was quantified with Image J. The graph shows the mean fluorescence intensity ±S.E.M. of 95 cells. *** $p<0.001$, Student's *t* test. Scale bar, 5 μm.

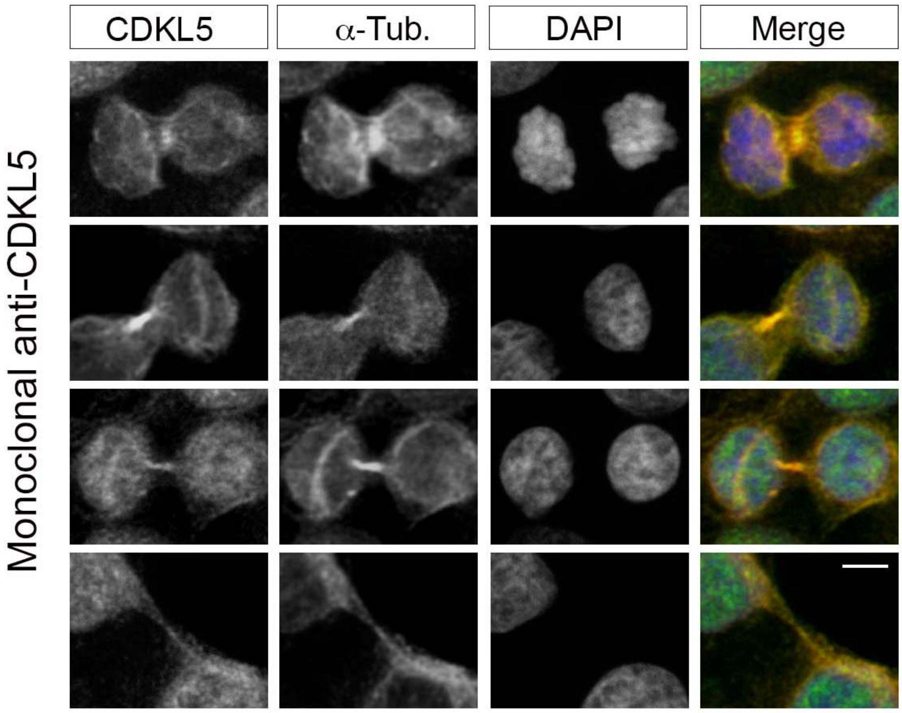

Supplementary Figure S2. Midbody localization of CDKL5 in HeLa cells can be detected also with a monoclonal anti-CDKL5 antibody. HeLa cells were stained for CDKL5 (monoclonal Ab, green), α-tubulin (red), and with DAPI (blue) to visualize DNA. Representative images of different stages of cytokinesis are shown. Scale bar, 5 μm.

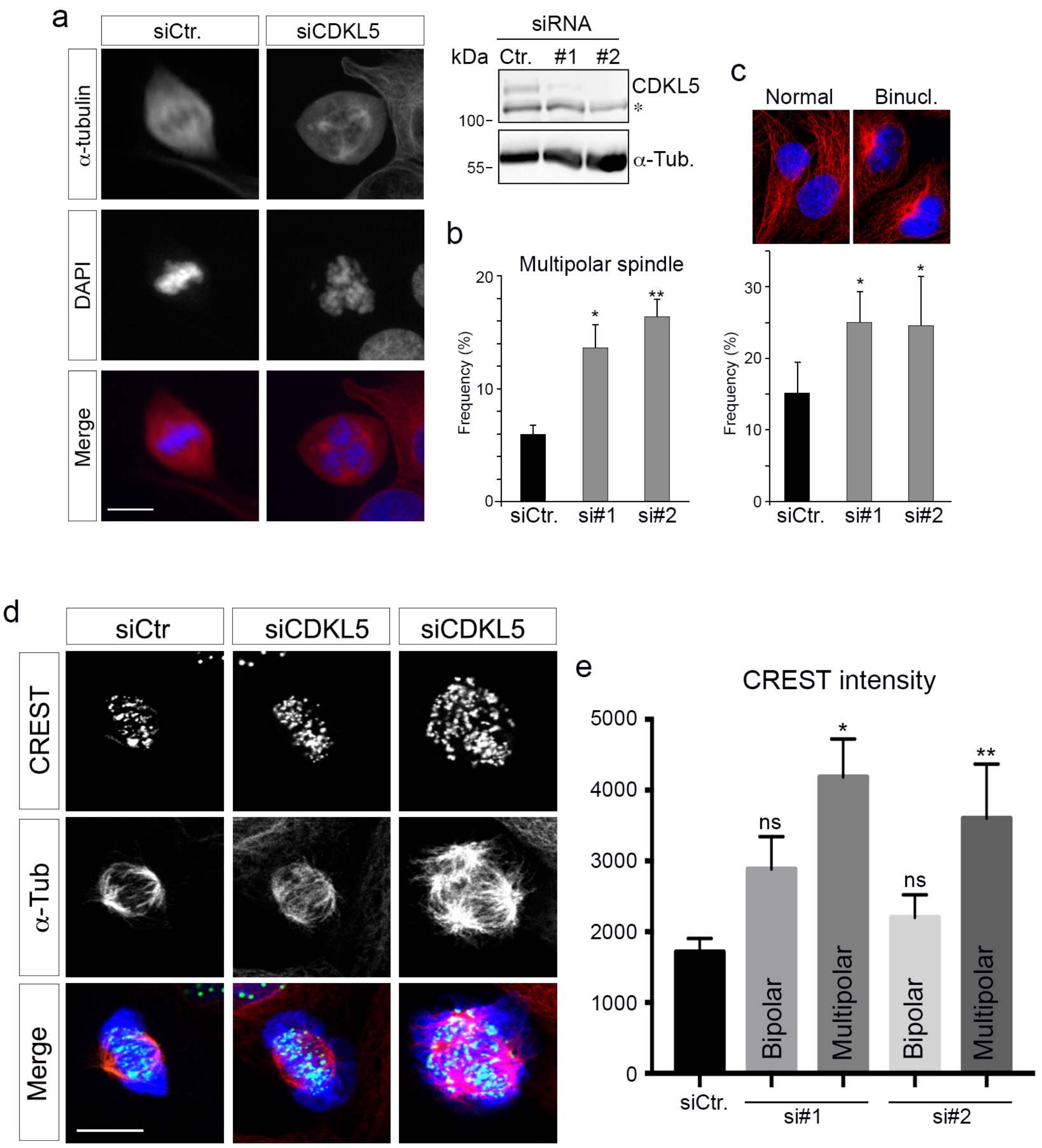

Supplementary Figure S3. Mitotic defects in MRC-5 cells silenced for CDKL5. **(a)** MRC5 cells were silenced for CDKL5 (si#1 or si#2) using siCtr as control. Silencing efficiency was verified by western blotting. Asterisk indicates an unspecific band recognized by the anti-CDKL5 antibody. **(b)** Graph showing the number of mitotic cells with multipolar spindles. Data represent mean ±SEM, *p<0.05; **p<0.01 in 4 independent experiments analyzing an average of 74 siCtr and 130 siCDKL5 cells in each experiment. Differences among groups were analyzed with Kruskall-Wallis and Dunn’s *post-hoc* test. **(c)** Graph showing the number of binucleated cells. Data represent mean ±SEM, *p<0,05; in 4 independent experiments analyzing an average

of 240 siCtr and 365 siCDKL5 cells in each experiment. Differences among groups were analyzed with Kruskall-Wallis and Dunn's *post-hoc* test. Representative image of normal and binucleated cells stained with anti-α-tubulin (red) and DAPI (blue) is shown. Scale bar, 10 µm. **(d)** MRC5 cells silenced for CDKL5 (si#1 and si#2) were stained for the centromeres (CREST; Antibodies Inc., 15-234-0001; green), α-tubulin (red), and with DAPI (blue). **(e)** Graph showing total CREST intensity in bipolar and multipolar mitosis. Data represent mean ±SEM, *$p<0.05$; **$p<0.01$ in 3 technical replicates analyzing 29 siCtr and 51 siCDKL5 cells. Differences among groups were analyzed with ANOVA and Dunnet's *post-hoc* test. CREST intensity was analyzed with ImageJ. Scale bar, 10 µm.

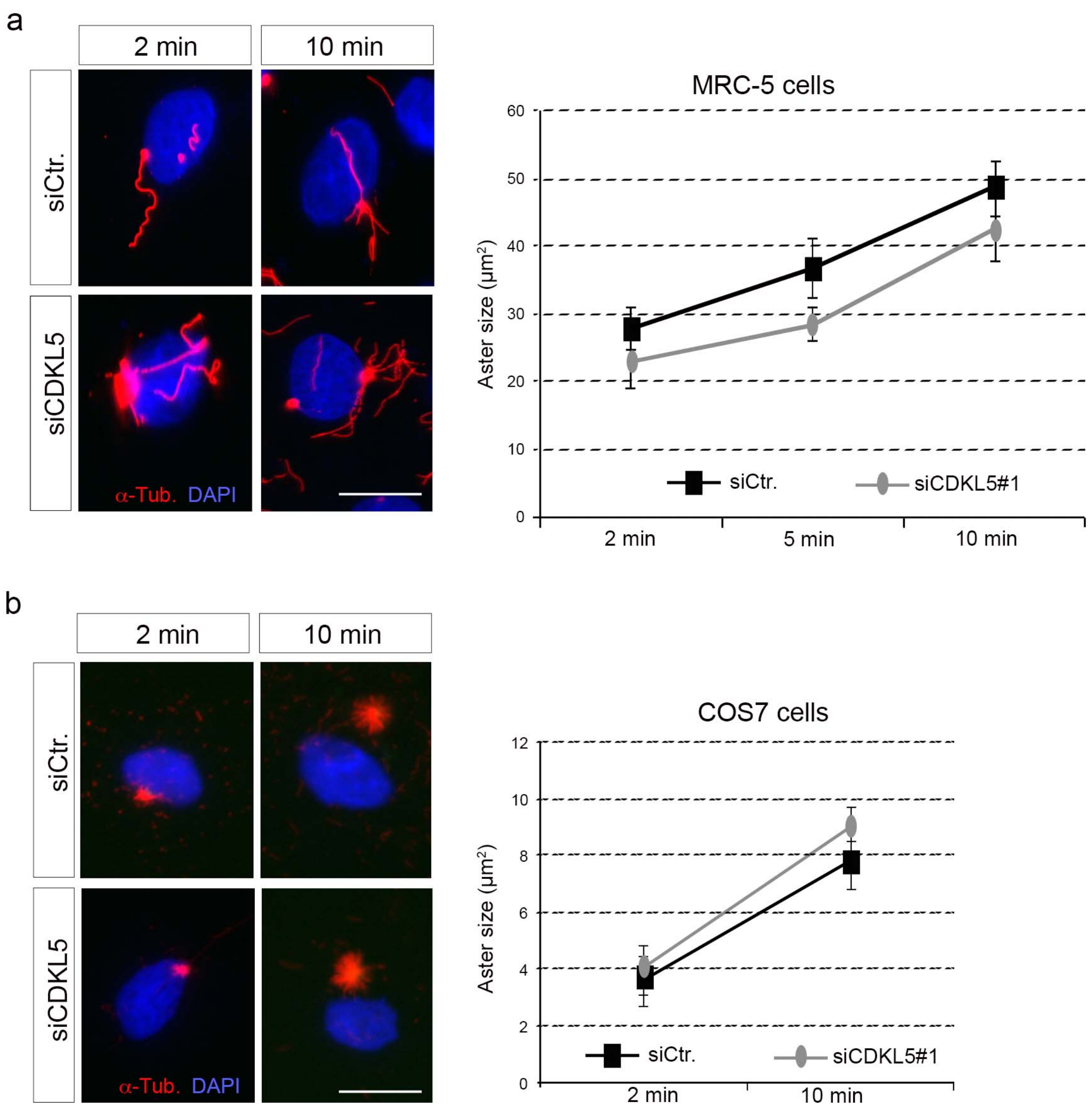

Supplementary Figure S4. CDKL5 deficiency does not affect microtubule nucleation. **(a)** MRC-5 and **(b)** COS7 cells were transfected with siCDKL5 or control siRNAs and the MT nucleation capacity was analyzed 60 h after silencing. MT disruption was obtained through incubation with 10 μg/ml nocodazole for 3 h. MT regrowth was tested by releasing cells in fresh media for 2, 5, and 10 min. Fixed cells were stained with an anti α-tubulin antibody and analyzed for the nucleation capacity through aster size. Scale bar, 10 μm.

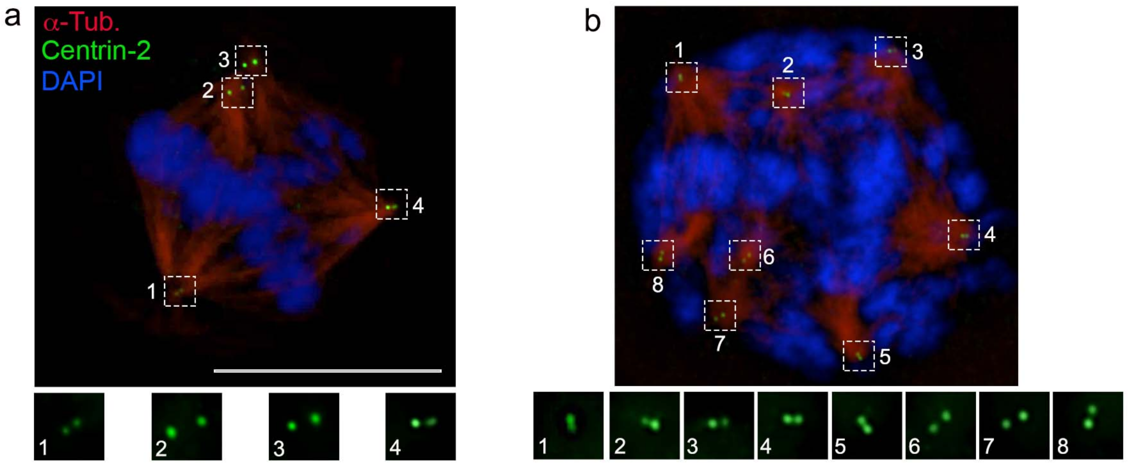

Supplementary Figure S5. Multipolar spindles of siCDKL5 cells show two centrioles at each individual pole. HeLa cells were silenced as in Figure 5a and analyzed after 60 h. Representative images of multipolar spindles in two distinct siCDKL5 cells are shown. Cells were stained with anti-β-tubulin (red) and anti-Centrin-2 (green) to mark spindles and centrioles, respectively. DAPI staining was used to visualize DNA. The spindle poles have been numbered and the insets below show the corresponding magnified centrin-2 signals (4X). Scale bar, 10 μm.

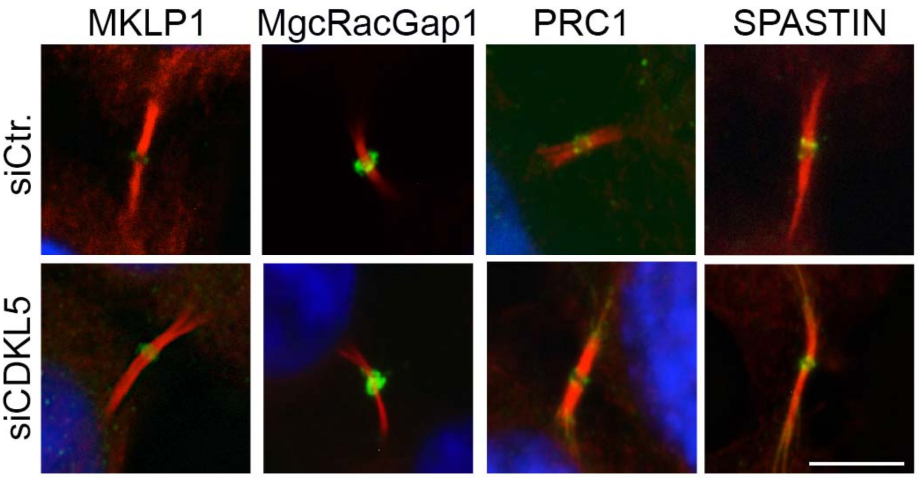

Supplementary Figure S6. CDKL5 deficiency does not interfere with midbody localization of MKLP1, MgcRacGap1, PRC1, or SPASTIN. HeLa cells were transfected with siCDKL5 or control siRNAs and stained for beta-tubulin (red) together with MKLP1, MgcRacGap1, PRC1, or SPASTIN (green). Scale bar, 10 μm.

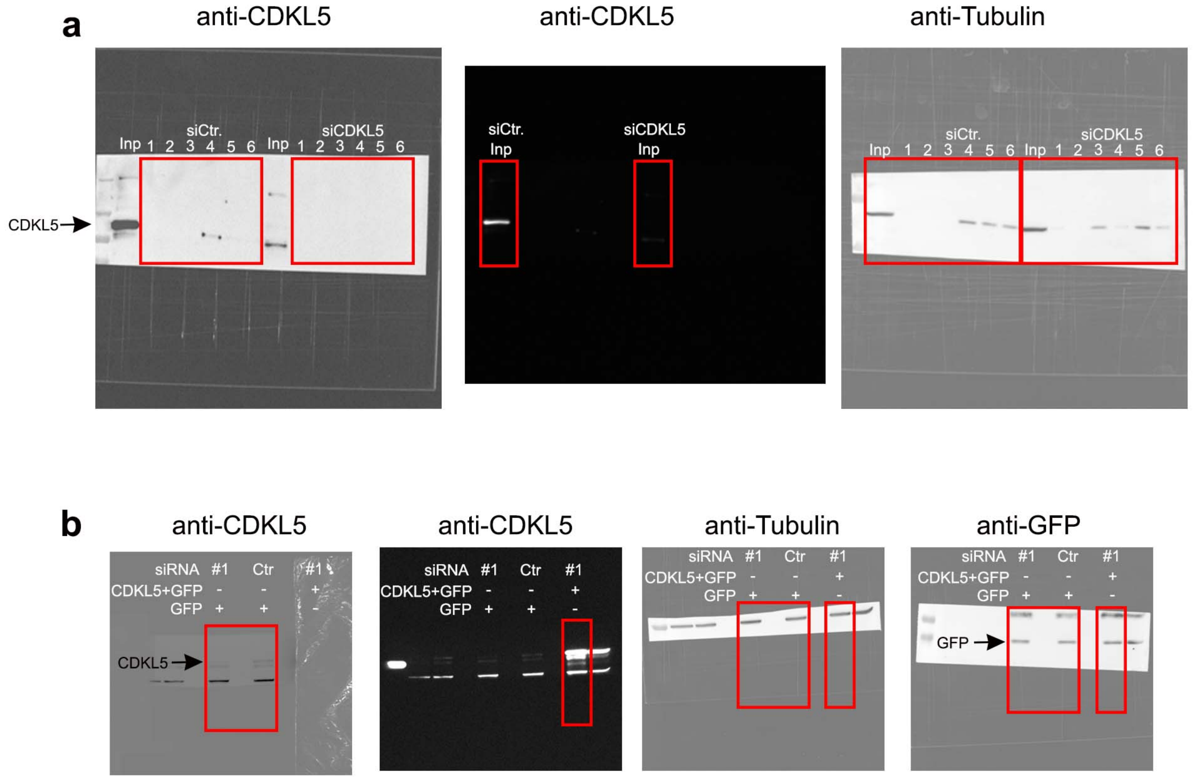

Supplementary Figure S7. Full-membrane blots of Figures 2c (**a**) and 4e (**b**). **(a)** Left membrane shows fractions 1-6 of HeLa cells transfected with siCtr and siCDKL5 as indicated. A lower exposure of the same membrane (middle panel) shows the Inputs, confirming the silencing of CDKL5. Right panel shows γ-tubulin levels. **(b)** The left-most membrane shows CDKL5 levels in HeLa cells transfected with siCtr and siCDKL5#1. The band corresponding to CDKL5 is indicated with an arrow. Please note that the membrane was turned 180° in Figure 4e and that the third lane with lysates overexpressing CDKL5 was covered during this exposure. The second membrane shows CDKL5 overexpression. Tubulin and GFP levels are shown in the two right panels.

Movie S1: Time-lapse Movie of asynchronous HeLa siCtr cells related to Figure 6a. DIC (differential interference contrast) images of cells were captured every 4 min. The display rate is one frame every 150 milliseconds. Still images of this video are shown in Figure **6b** upper panels.

Movies S2: Time-lapse Movie of Asynchronous HeLa siCDKL5 cells Related to Figure 6a. DIC images of cells were captured every 4 min. The display rate is one frame every 150 milliseconds. Still images of this video are shown in Figure **6b** middle panels.

Movies S3: Time-lapse Movie of Asynchronous HeLa siCDKL5 cells Related to Figure 6a. DIC images of cells were captured every 4 min. The display rate is one frame every 150 milliseconds. Still images of this video are shown in Figure **6b** lower panels.
